# A Microfluidic, High Throughput Protein Crystal Growth Method for Microgravity

**DOI:** 10.1371/journal.pone.0082298

**Published:** 2013-11-21

**Authors:** Carl W. Carruthers Jr, Cory Gerdts, Michael D. Johnson, Paul Webb

**Affiliations:** 1 Houston Methodist Research Institute, Department of Genomic Medicine, Houston, Texas, United States of America; 2 Protein BioSolutions, Inc., Gaithersburg, Maryland, United States of America; 3 NanoRacks LLC, Houston, Texas, United States of America; University of South Florida College of Medicine, United States of America

## Abstract

The attenuation of sedimentation and convection in microgravity can sometimes decrease irregularities formed during macromolecular crystal growth. Current terrestrial protein crystal growth (PCG) capabilities are very different than those used during the Shuttle era and that are currently on the International Space Station (ISS). The focus of this experiment was to demonstrate the use of a commercial off-the-shelf, high throughput, PCG method in microgravity. Using Protein BioSolutions’ microfluidic Plug Maker™/CrystalCard™ system, we tested the ability to grow crystals of the regulator of glucose metabolism and adipogenesis: peroxisome proliferator-activated receptor gamma (apo-hPPAR-γ LBD), as well as several PCG standards. Overall, we sent 25 CrystalCards™ to the ISS, containing ~10,000 individual microgravity PCG experiments in a 3U NanoRacks NanoLab (1U = 10^3^ cm.). After 70 days on the ISS, our samples were returned with 16 of 25 (64%) microgravity cards having crystals, compared to 12 of 25 (48%) of the ground controls. Encouragingly, there were more apo-hPPAR-γ LBD crystals in the microgravity PCG cards than the 1g controls. These positive results hope to introduce the use of the PCG standard of low sample volume and large experimental density to the microgravity environment and provide new opportunities for macromolecular samples that may crystallize poorly in standard laboratories.

## Introduction

Biochemical macromolecules are fundamental components of all living things. Understanding a macromolecule’s three-dimensional structure provides a deeper understanding of its function and relationship to other components that are responsible for maintaining life. Throughout the field’s history, structural biology has been a leading contributor to the areas of biotechnology, pharmaceuticals, and academia. For example, efforts with recent structural data from G protein coupled receptors holds promise for novel treatments for cardiovascular disease, obesity and cancer [1,2].

Previously, it required years of expensive laboratory and computational effort to create a three dimensional macromolecular model using X-ray crystallography. A small number of options were available for the expression of target proteins and relatively few conditions were screened to determine solubility and crystal growth. Completion of the Human Genome Project in 2003 led to the formation of large structural genomic programs such as the NIH’s Protein Structure Initiative, Japan’s RIKEN and the Structural Genomics Consortium. These collaborative “structure factories” have been crucial in reducing the cost of determining a macromolecular model as well as driving the production of effective technology and methodologies [3,4]. Some of their accomplishments include: more efficient cloning, expression, and purification methods; low volume, high throughput screening for solubility and crystal growth; fluid handling robots; and computational programs for collecting data and solving structures. These developments have quickly altered the landscape of pharmaceutical and academic structure laboratories, allowing for unprecedented contributions to the body of structural knowledge [5-7]. 

Despite these advances, there continue to be areas needing improvement. A recent publication cites that of 125,316 genes cloned for a structural genome project, only 6.9% led to structural models, with most of the failures arising from an absence of diffraction quality crystals [7]. One possibility to improve this statistic is to utilize microgravity to increase the yield of quality crystals. As a crystal forms on Earth it depletes the surrounding solution of protein, creating areas of lower density. Because of this, buoyancy driven convection results in the growing crystal rising and falling in the crystallographic solution, inducing uneven growth rates. Another concern in terrestrial growth conditions is sedimentation. As a crystal becomes larger, its increasing mass causes it to settle against a drop’s liquid/air interface (i.e. hanging drop method) or a growth chamber wall. This orientation can prevent consistent three-dimensional growth and may lead to distortions in the crystal. Acting together, these effects create a highly dynamic environment that can cause imperfections in a crystal lattice. In microgravity, buoyant convection and sedimentation are negligible; therefore crystals move very little and grow at a more uniform rate, which may result in a better quality crystal [8-12].

Microgravity protein crystal growth (μg PCG) experiments flown on NASA’s Space Shuttle between 1983 and the early 2000’s provided evidence that it is feasible to grow crystals that demonstrate improved diffraction, increased signal to noise, and/or lower mosaicity [8] compared to those grown under 1g conditions. Often cited examples of these results are lysozyme [13-17] and insulin [18,19] while there exist almost two dozen other microgravity protein structure examples currently deposited in the Protein Data Bank [8]. Some of these pharmacologically important successes include: the crystallization and increased diffraction quality of the proto-oncogene EGF receptor (EGFR/HER1) which led to the first time a space group could be determined for this important protein [20]. A crystal grown in microgravity of the antibiotic target and metabolically important NAD^+^ synthetase provided improved data sufficient to propose a never seen before second catalytic step as well as the design of new drug targets [21]. Finally, optimal microgravity crystallization conditions for the large dimeric, multidomain aminoacyl-tRNA synthetase were obtained over the course of several spaceflights. This iterative process resulted in crystals with superior intensity, lower mosaicity and higher resolution [22,23] than previously obtained.

During the Space Shuttle era a variety of unique devices were created for growing and studying the μg PCG process [24-31]. Yet, while a technological revolution was occurring in terrestrial labs during the 2000’s, the evolution of microgravity PCG technology stalled. With the retirement of the Space Shuttle program in 2011 and the International Space Station (ISS) being declared “open for business” [32], commercial companies such as NanoRacks, LLC or the non-profit Center for the Advancement of Science in Space (CASIS) are currently providing unprecedented research access to the ISS. Realizing there is a clear need to revitalize microgravity crystallography research with current technology and methods, we partnered with NanoRacks to demonstrate a commercial off-the-shelf (COTS) candidate for μg PCG. In comparison with usual 1g labs, Shuttle era microgravity PCG devices require large volumes of protein (100’s of μL) and typically allow for only a single sample parameter in each well. In contrast, Protein BioSolutions’ Plug Maker™ [33,34] requires a small volume of protein (~4 μL), that enables testing of a large experimental sample space (400-800 individual experiments) in a self-contained card the size of a microscope slide. Because of these parameters, the Plug Maker™/CrystalCard™ system seemed like an excellent COTS choice to test for μg PCG. 

The purpose of this pilot experiment was to test PCG using the Plug Maker™/ CrystalCard™ system in a microgravity environment. Despite their importance, there are only a few unbound structures of nuclear receptors available and knowledge of their 3D structure is critical to understanding receptor-ligand and co-regulator interactions, as well as bound allosteric effects [35,36]. Using the unbound ligand binding domain of the metabolically important nuclear receptor nuclear receptor peroxisome proliferator-activated receptor gamma (apo-hPPAR-γ LBD) and five model proteins, we set up cards in a variety of protein, buffer and precipitate conditions around their usual terrestrial crystal growing conditions. Once demonstrated that crystals can be obtained using this method, we hypothesize that using µg crystals grown on future flights will decrease the mosaicity of our current apo-hPPAR-γ LBD structure, leading to a better quality model. The additional control proteins for this project were chosen because of their experimental history as standards for 1g and μg PCG as well as presenting differing levels of crystallization difficulty. 

In coming years, as NASA, CASIS and commercial companies create reliable, more cost effective access ISS National Lab facilities, we feel a successful method like this could supplement the number of macromolecular structures acquired or improve existing data sets, creating more opportunities for academic and pharmacological discoveries.

## Materials and Methods

### Proteins Used for Crystallization

The apo ligand binding domain (amino acids 315-505) of hPPAR-γ was expressed from pET28a transformed into *E. coli* BL21 (DE3). Transformed bacteria were grown in 3 L SOB media and induced at an OD_600_ = 0.8 with 0.750 mM IPTG/1.5 L for 18h at 18 °C. After centrifugation and decanting of media, bacterial pellets were re-suspended in 50mM Tris pH 7.4, 500 mM NaCl, 20 mM imidazole, and 20 mM β-mercaptoethanol. The cell suspension was sonicated on ice until viscosity was reduced. Cell lysate was applied to a 5 mL IMAC HiTrap FF charged with nickel, washed until A_280_ returned to baseline, and then eluted with a gradient of 50 mM Tris pH 7.4, 500 mM NaCl, and 500 mM imidazole. Eluted sample was concentrated and applied to a HiPrep 26/10 desalting column equilibrated with 50 mM Tris pH 8.0, 10 mM NaCl. Sample was then applied to a 6 mL RESOURCE Q column and eluted with a gradient of 50 mM Tris pH 8.0, 1M NaCl. Eluted sample was concentrated to 15 mg/mL, sterilized using a 0.22 μm filter, aliquoted and frozen in liquid nitrogen until needed.

Lyophilized chicken egg white lysozyme (Sigma-Aldrich, St. Louis, MO. Catalog number L6876) was re-suspended in 0.1 M sodium acetate pH 4.5 to a final concentration of 100 mg/mL. Glucose isomerase, lipase B, xylanase, and thermolysin were purchased from Hampton Research (Aliso Viejo, CA. Catalog numbers HR7-100, HR7-099, HR7-104 and HR7-098, respectively) and prepared as stated in supplier’s instructions. All samples were sterilized using a 0.22 μm filter and snap frozen in liquid nitrogen until needed. 

### Initial Concentrations of Precipitants

Apo-hPPAR-γ LBD: 2.0 M sodium citrate; chicken egg white lysozyme: 1.1 M NaCl; glucose isomerase: 2.5 M ammonium sulfate pH 7.0; lipase B: 20% 2-propanol, 20% PEG 3350, 0.1 M sodium acetate pH 5.5; thermolysin: 1.5 M ammonium sulfate, 12% glycerol, 0.1 M Tris pH 8.5; xylanase: 1.0 M ammonium sulfate, 0.1 M sodium citrate pH 4.3.

### Filling and Freezing of CrystalCards™

All frozen protein, buffer and precipitant samples were shipped overnight on dry ice to the Emerald Bio facility at Bainbridge Island, WA. Upon thawing, glucose isomerase was observed to have precipitated and therefore was not used. The CrystalCards™ were filled using Protein BioSolutions, Inc.’s (Gaithersburg, MD) Plug Maker™ with the parameters provided in Table 1. Two cards of each protein sample were made, one for microgravity and one as a 1g control, for a total of 50 cards (25 for flight and 25 for ground controls). After each card was filled (~30 seconds) it was immediately submerged in liquid nitrogen and then placed in a microscope slide box on dry ice until all cards were filled. Afterwards, all cards were stored at -80 °C. 

**Table 1:** Protein Samples, Plug Maker Parameters and Crystallization Results.

			**Plug Maker Flow Rate (μL/minute)**									
	**Sample #**		**Protein**		**Buffer**		**Precipitate**		**Carrier Fluid**		**Crystals?**	
**Protein**	**μg**	**1g**	**Start**	**Finish**	**Start**	**Finish**	**Start**	**Finish**	**Start**	**Finish**	**μg**	**1g**
Lipase B	989	990	2.0	2.0	0.5	1.5	2.0	1.0	5.0	5.0	N	N
	991	992	3.0	1.0	0.5	0.5	1.0	3.0	5.0	5.0	N	N
	1015	1016	2.0	2.0	0.5	0.5	2.0	2.0	0.1	0.1	N	N
Xylanase	993	994	2.0	2.0	0.5	0.5	2.0	1.0	6.5	5.5	Y	N
	995	996	3.0	1.0	0.5	0.5	1.0	3.0	5.5	5.5	N	N
	997	998	2.0	2.0	0.5	0.5	2.0	2.0	8.0	1.5	N	N
	1027	1028	2.0	2.0	0.5	0.5	2.0	2.0	0.1	0.0	N	N
	1029	1030	2.0	1.0	0.5	1.5	2.0	2.0	5.5	5.5	N	N
Lysozyme	999	1000	2.0	2.0	0.5	1.5	2.0	1.0	5.5	5.5	Y	Y
	1001	1002	3.0	1.0	0.5	0.5	1.0	3.0	5.5	5.5	Y	Y
	1003	1004	2.0	2.0	0.5	0.5	2.0	2.0	8.0	1.5	Y	Y
	1005	1006	2.0	2.0	0.5	0.5	2.0	2.0	0.5	0.5	Y	Y
	1031	1032	2.0	1.0	0.5	1.5	2.0	2.0	5.5	5.5	Y	Y
	1033	1034	2.0	2.0	0.1	2.0	2.0	0.1	0.1	0.0	Y	Y
apo-hPPAR-γ LBD	1017	1018	1.0	3.0	0.5	0.5	3.0	1.0	8.0	8.0	Y	N
	1019	1020	1.0	2.0	1.5	0.5	2.0	2.0	8.0	8.0	Y	N
	1021	1022	2.0	2.0	0.5	1.5	2.0	1.0	8.0	8.0	Y	N
	1023	1024	2.0	2.0	0.5	0.5	2.0	2.0	0.1	0.0	N	N
	1025	1026	1.0	3.0	0.5	0.5	3.0	1.0	8.0	8.0	Y	N
	1035	1036	2.0	2.0	0.1	2.0	2.0	0.1	0.1	0.0	Y	Y
	1037	1038	5.0	0.1	0.5	0.5	0.1	5.0	0.1	0.0	N	Y
Thermolysin	1007	1008	2.0	2.0	0.5	1.5	2.0	1.0	5.5	5.5	Y	Y
	1009	1010	1.0	3.0	0.5	0.5	3.0	1.0	5.5	5.5	Y	Y
	1011	1012	2.0	2.0	0.5	0.5	2.0	2.0	8.0	1.5	Y	Y
	1013	1014	2.0	2.0	0.5	0.5	2.0	2.0	0.1	0.1	Y	Y

### Storage and Transport to the ISS

**Figure 1:**
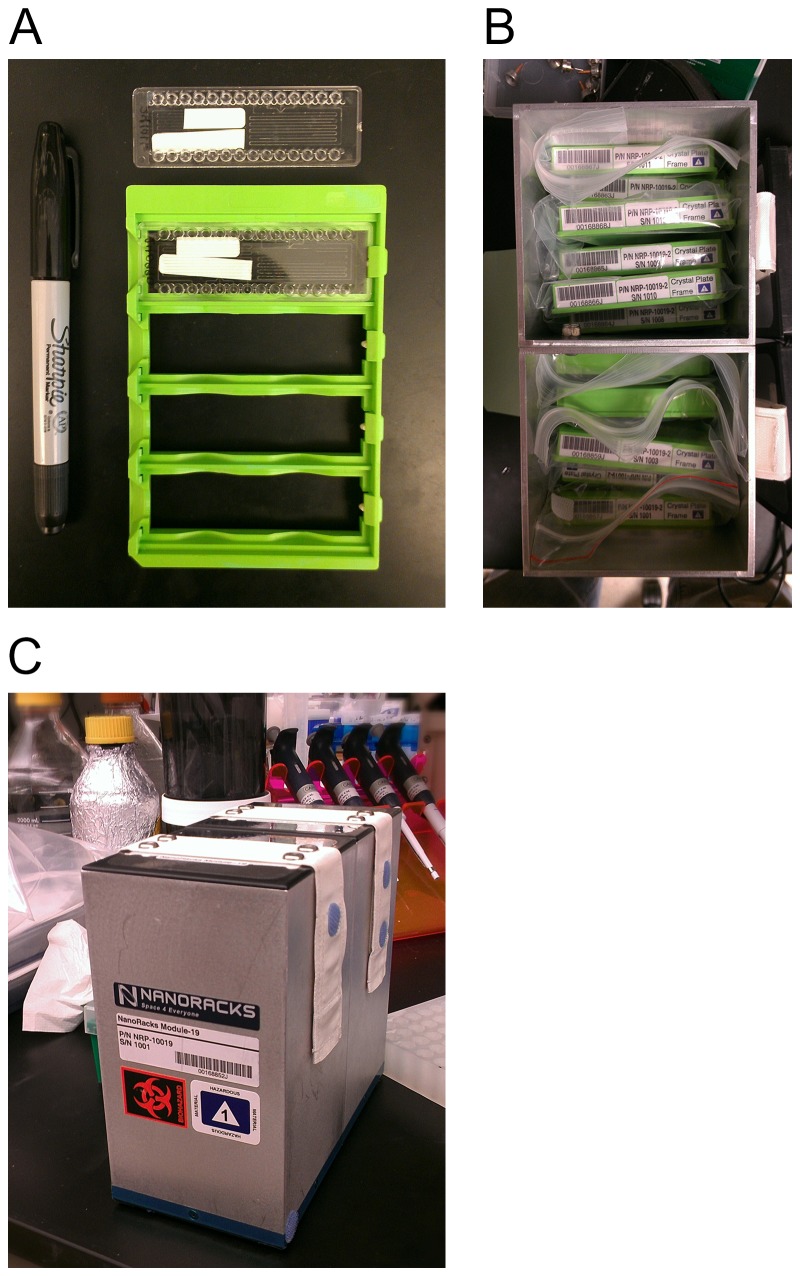
CrystalCards™, Frames and Packaging of NR PCG1. A) CrystalCard™ alone (top) and CrystalCard™ in frame (bottom); B) Frames containing CrystalCards™ stored vertically in two attached 1.5U NanoRacks NanoLabs; C) The fully assembled NanoRacks NanoLab NR PCG1.

Frozen samples were shipped overnight on dry ice from the Emerald Bio facility to The Houston Methodist Research Institute in Houston, Texas where they were stored at -80 °C. Ten days before launch all cards were placed in card frames, inserted into individual zip closure plastic bags and placed into two attached 1.5U NanoRacks NanoLab modules (called NR PCG1, see Figure 1) and shipped overnight in dry ice to the SpaceX launch facility at Kennedy Space Center, Florida. NR PCG1 was kept at -80 °C until moved to the -95 °C General Laboratory Active Cryogenic International Space Station (ISS) Experiment Refrigerator (GLACIER) on the SpaceX Dragon capsule ~12 hours before launch. Launch occurred on March 1, 2013 at 15:10 UTC. On March 4, 2013 at 19:00 UTC, NR PCG1 was removed from the GLACIER and stowed in Expedite the Processing of Experiments for Space Station (EXPRESS) rack 4 on the Japanese Experiment Module of the International Space Station. The experiment was allowed to free float and thaw at ambient station temperature (23-24 °C) while in the EXPRESS rack locker.

An identical NanoLab module was filled with the ground control sample cards and treated to the same changes in temperature (dry ice, -80 °C) as flight samples. Controls were removed from -80 °C on March 4, 2013 and allowed to thaw in a 24 °C incubator. Samples were stowed in the incubator at that temperature until the end of the experiment and only removed for surveying.

### On-Orbit Microscope Survey of CrystalCards™

Cards were surveyed on the ISS while in their frames on April 29, 2013 ~09:00 UTC (56 days) using NanoRacks’ USB microscope (Celestron 2MP Handheld Digital Optical Microscope, #44306). About 7-8 pictures were taken of each slide with the microscope at low magnification. A second survey of a single slide at high magnification while removed from the frame occurred on May 8, 2013 ~10:00 UTC (65 days). 

### Return of Samples from the ISS

After 70 days of exposure to microgravity, the samples returned aboard Soyuz TMA-07M, undocking from the ISS on May 13, 2013, 23:08 UTC and landing in southern Kazakhstan on May 14, 2013 at 02:31 UTC. The samples were documented again upon arrival in Houston, Texas on May 15, 2013 06:00 UTC (27.5 hours after returning to Earth). During the return journey the microgravity samples were subjected to high g-forces several times and multiple temperature changes: ~24 °C on the ISS, max ~31-32 °C on Soyuz during re-entry, ~10 °C at the landing area, unknown return transport helicopter and aircraft temperatures, ~22 °C during transport to the lab and 22 °C at the lab. The 1g control samples were also reviewed at this time also.

## Results and Discussion

### Filling and Freezing of Plug Maker™ CrystalCards™

Similar to other available high-throughput fluid handling devices (e.g. TTP Labtech's Mosquito®, Art Robbins Instruments’ Gryphon, etc.) the Plug Maker™ system provided a straightforward, process of creating a large variety of conditions for PCG using about 10 nL of protein per plug (~2-4 μL/card). The parameters chosen for each protein were determined to test if variations of the four solutions (protein, buffer, precipitate and carrier fluid) would produce crystals of different size or morphologies than those in 1g.

Previously, almost all methods of μg PCG required samples to be stable in solution and was subjected to weeks of storage before loading, launch and travel to the microgravity environment. This prerequisite decreased opportunities for the investigation of proteins that are unstable in solution for long periods of time. Freezing of the filled CrystalCards™ was advantageous in making it possible to store the samples at -80 °C indefinitely, increasing our experiment flexibility with uncertain or scrubbed/aborted launches. This method may exclude proteins that cannot tolerate being frozen. It may therefore be beneficial on future projects to experiment with temperature gradient PCG, which has been shown to work well in microgravity [8,23], using the Plug Maker™ method.

### On-Orbit Microscope Survey of CrystalCards™

**Figure 2:**
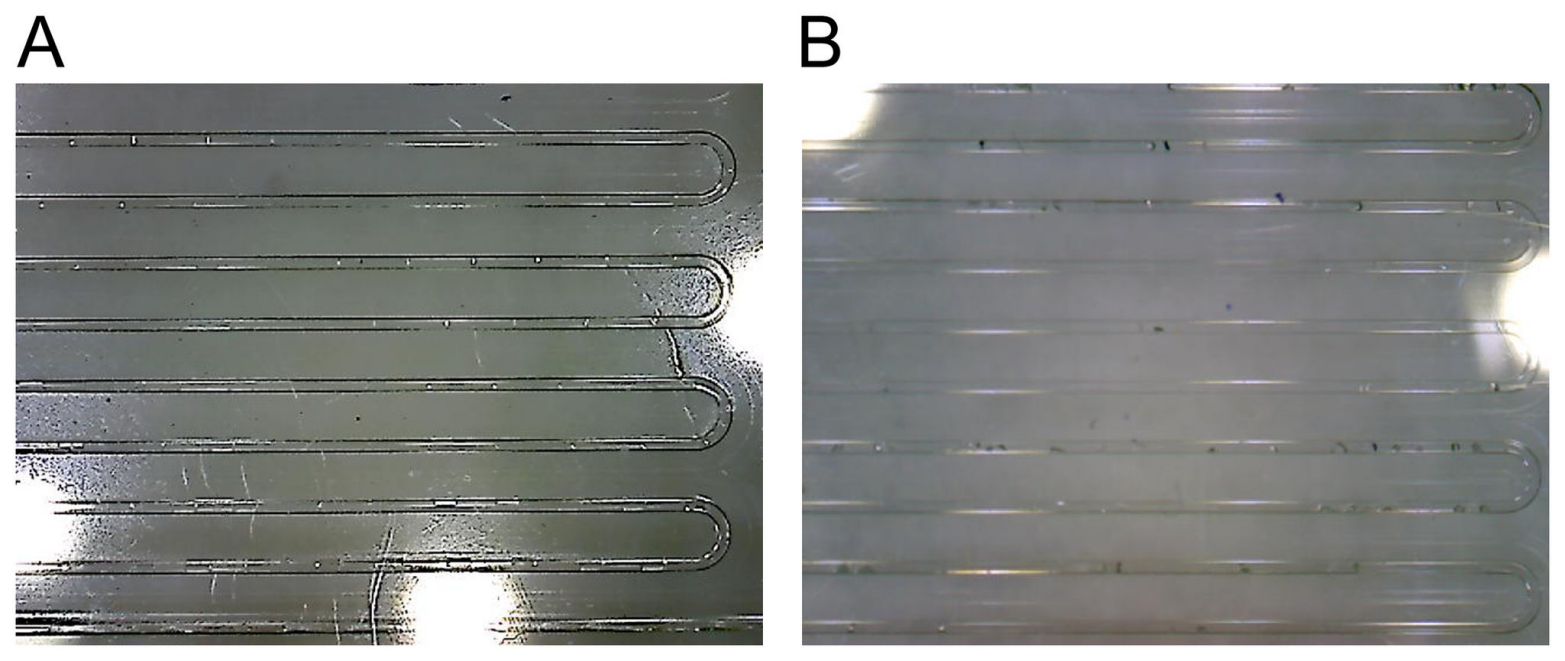
Comparison of Low Magnification Pictures Taken with USB Microscope. An example of one of the survey pictures taken with the USB microscope at low magnification taken while in orbit (A) and with an identical USB microscope on the ground using the same setting of the corresponding control card (B). While still difficult, it was much easier to see possible crystals in the pictures taken with the USB microscope of the ground control card than of those pictures returned to us from the ISS.

The first survey of all 25 cards was completed with the USB microscope accidently set at the low magnification setting (Figure 2A). While the astronaut was performing the survey, it was not apparent if the microscope was in the correct (high-magnification) mode and this error was not discovered until ground acquisition of the data several hours later. Furthermore, for unknown reasons, the quality of the images received from the ISS was very poor compared to our 1g photographs taken by an identical microscope at the same low magnification setting (Figure 2B). Unfortunately, at this magnification and data quality the microgravity images were not sufficient to conclusively determine if crystals were present.

Future Plug Maker™ μg PCG flights will have a more thorough protocol to clarify the correct microscope setting to prevent future misunderstandings, also possibly incorporating an automated surveying process. While it is not absolutely necessary to observe the crystals on orbit, doing so provides evidence that the crystals were grown in microgravity and not on the return journey to the lab. *In situ* observation would also be important in determining if the crystals have reached a sufficient size for return or if more time in orbit is required.

**Figure 3:**
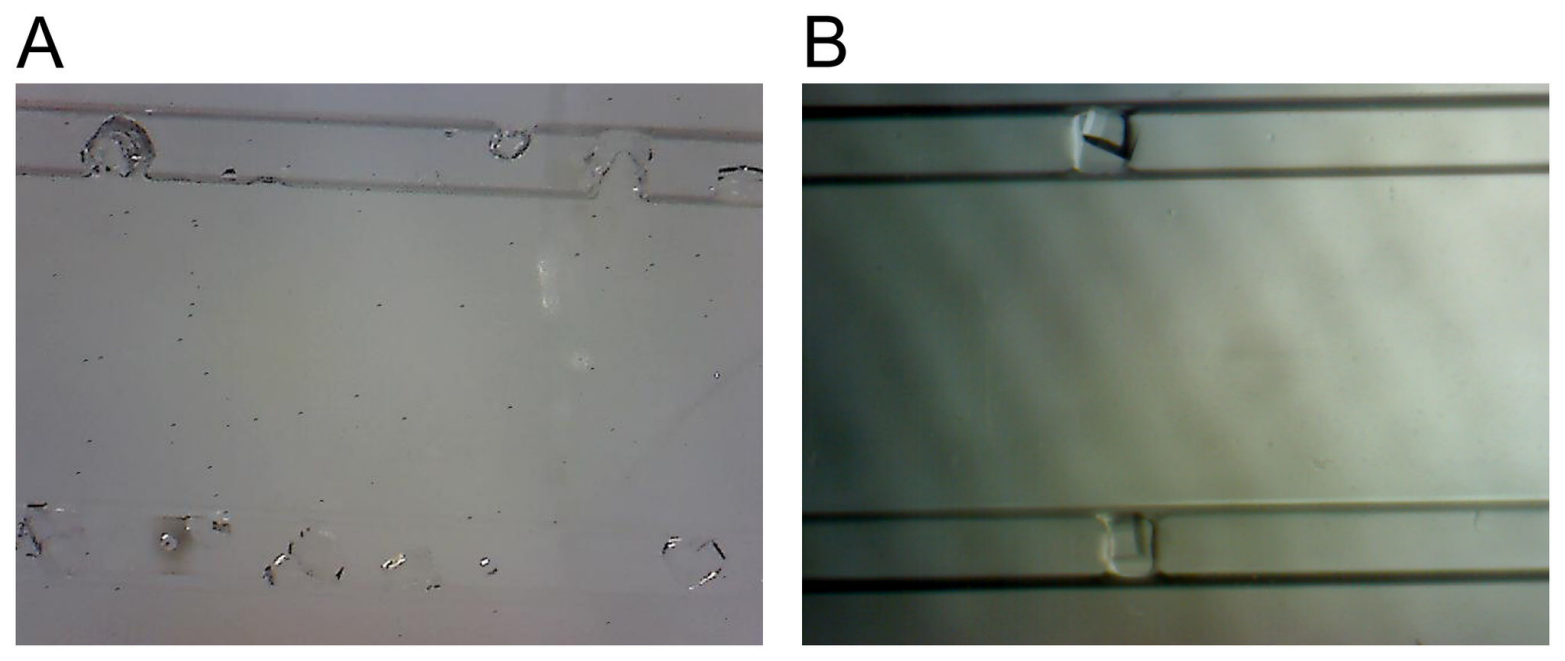
Examples of pictures taken during the second survey with the USB microscope set to high magnification. (A) High magnification picture of lysozyme crystals grown in microgravity and (B) high magnification picture of lysozyme crystals grown in the ground control samples.

While gaining impromptu access to tightly scheduled astronaut time is not easy, due to the coordination provided by NanoRacks we were granted time a week later to document one lysozyme sample card (Table 1, #1033). This second survey was completed using the microscope’s high magnification setting and allowed us to conclude that crystal growth had occurred in microgravity for this card. Upon examination of these pictures (Figure 3A), the lysozyme crystals appeared to vary in size and quantity comparable to pictures of the corresponding 1g control card (Table 1, #1034) with many ~100-150 μm crystals found in both. Because crystals were observed in other 1g control protein samples, we feel confident in assuming that crystal growth of the microgravity samples had occurred on the ISS as well.

### Crystal Samples After Returning to Earth

During the 31 hour trip from the ISS to the lab in Houston, Texas, the microgravity samples were subjected to high g-loads (possibly up to 9g’s) as well as multiple climate conditions. Despite the absence of temperature control, numerous crystals were observed in these samples. Inspection of the cards after their return revealed that 16 of the 25 microgravity and 12 of the 25 ground control CrystalCards™ had crystals present (Table 1). None of the cards or frames appeared damaged and no signs of leakage were present. Although future Plug Maker™ μg PCG units will be adapted with active temperature control, these results emphasize the robustness of this relatively simple PCG method. 

### Comparison of Microgravity and Ground Control Crystals

#### Human apo-PPAR-γ LBD

**Figure 4:**
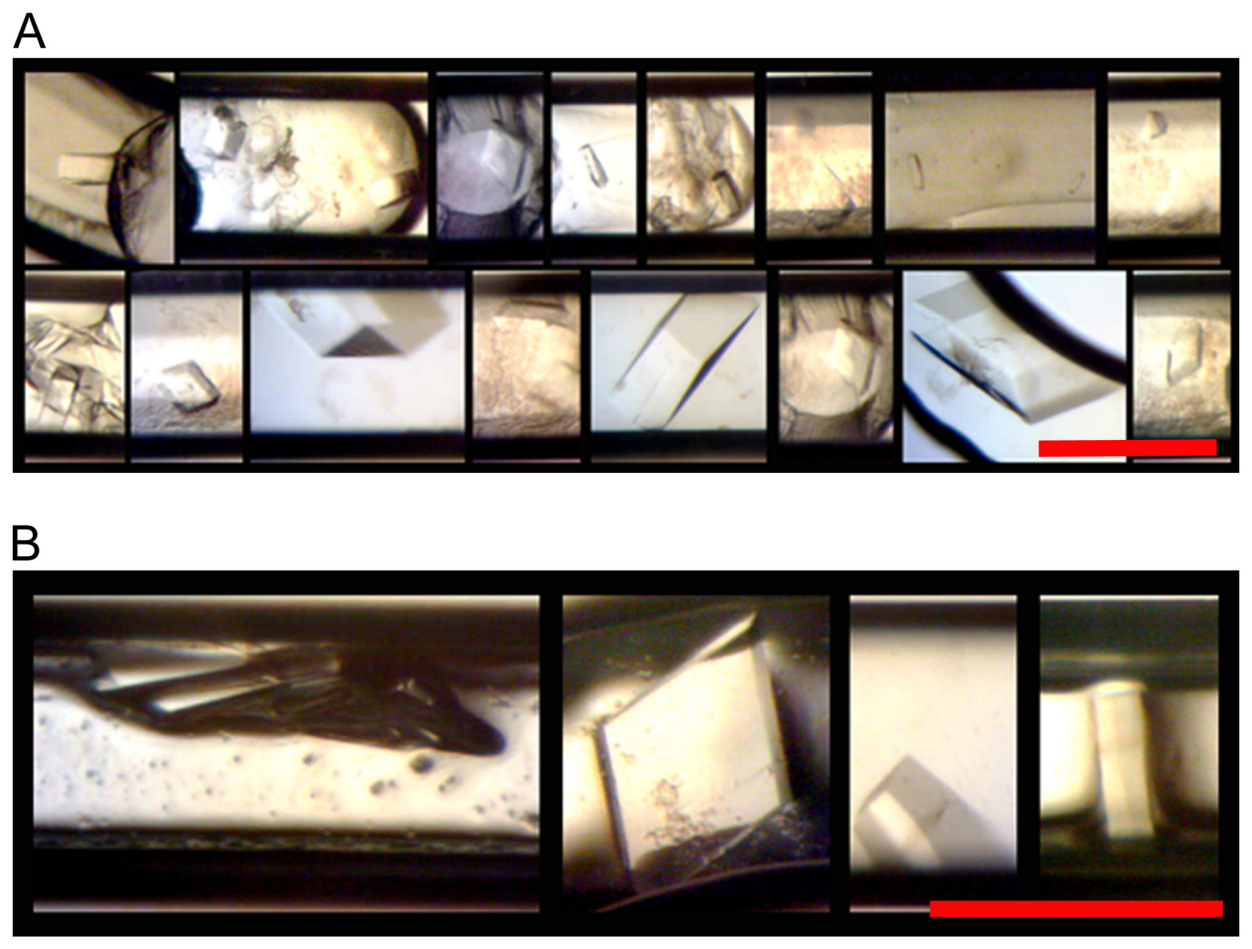
Examples of apo-hPPAR-γ LBD Crystals. (A) Returned from microgravity and (B) ground control crystals. Red bar indicates 200 μm.

Five out of seven apo-hPPAR-γ LBD cards flown in microgravity formed single crystals. At 200+ μm, the three largest crystals were from microgravity card number 1035 with the other microgravity crystals being ~100 μm. The corresponding ground control of 1035, 1036, was one of only two ground control cards that had crystals (see Figure 4). The largest and visually deformation free crystals were from the microgravity cards that kept the protein concentration constant with non-uniform plug sizes. As is common with apo-PPAR-γ LBD crystals grown in our lab, the majority of the plugs had skin and/or heavy, amorphous precipitate. 

Since apo-hPPAR-γ LBD is not a model protein, the growth of these crystals in microgravity is very encouraging. This member of the nuclear receptor family has been shown to play roles in glucose and lipid metabolism [35]. The function of nuclear receptors in gene regulation, as well as the hydrophobic pocket of their ligand binding domains, naturally make them candidates for drug design and highly sought after disease regulation targets [36]. However, challenges in forming high quality apo and full length structures of nuclear receptors, such as hPPAR-γ have led to uncertainties when discriminating between possible endogenous ligands from bacterial contaminants during purification [37], possibly creating an overly simplistic view of their allosteric mechanisms [38]. Success with these samples may encourage other researchers to optimize their therapeutic target structures using μg PCG.

Samples of apo-hPPAR-γ LBD were flown in 2011 on the last two space shuttle missions (STS-134 and STS-135) using another μg PCG method that required much larger volumes of protein/precipitant and which also had ambient temperature control. Both flights returned with only amorphous precipitate and no exact cause for the failure was determined. Possible reasons were temperature fluctuations during loading and/or delivery to the orbiter, unsteady orbiter cabin temps, etc. It was also not possible to determine if the device was activated properly or if crystals may have grown in orbit, but melted on the return trip to the lab. Because of our previous shuttle results and the fact that NR PCG1 was also passively temperature controlled, we did not anticipate having any crystals returned to us and therefore decided only to test if this method will work for μg PCG before committing to testing for diffraction quality. In light of our positive results and since subsequent flights using this method will have active temperature control, we plan on flying these same samples again using the optimized conditions from this experiment and then examine the microgravity grown crystals for better diffraction quality/lower mosaicity.

#### Lysozyme

**Figure 5:**
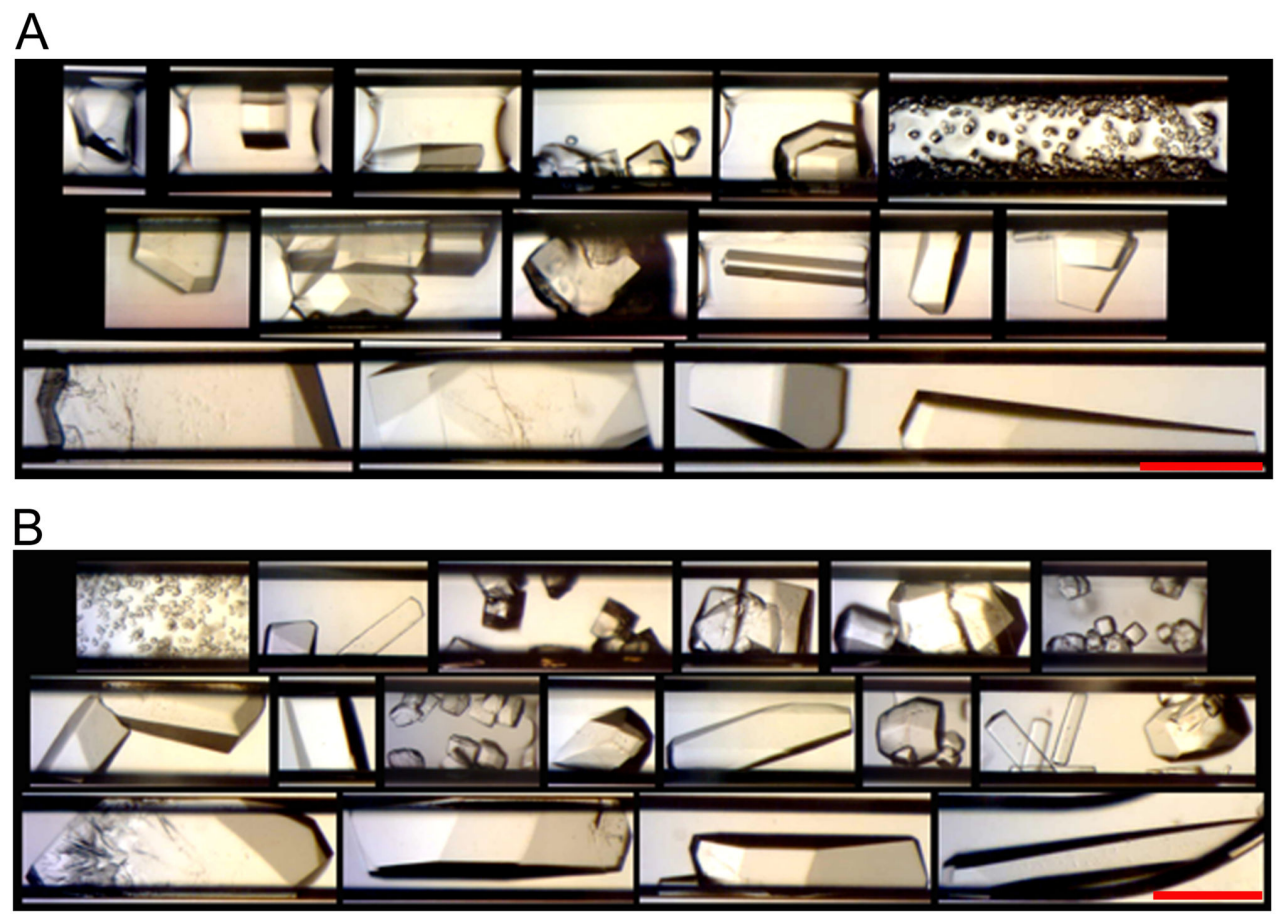
Examples of Lysozyme Crystals. (A) Returned from microgravity and (B) ground control crystals. Red bar indicates 200 μm.

Lysozyme has been shown to grow very well in microgravity [13-17]. For this experiment, lysozyme had the greatest number, variety of crystal size and morphology of all the protein samples. Due to the large number of crystals (each card had hundreds) it was not possible to compare them all. Figure 5 shows representatives of crystals from the microgravity and ground control samples.

Cards numbered 1033 and 1034 produced the most single large (300-450 μm x 150-200 μm) crystals with cards 1031 and 1032 as the second best. The remaining four cards had either showers or clusters of small crystals ranging from 10 μm to 50+ μm. While there seemed to be slightly more large crystals in the microgravity samples, overall, the microgravity cards appeared to be similar to their corresponding ground controls.

The difference between these cards is that 1033/1034 had a constant protein concentration, with varied buffer, precipitate and carrier fluid flow rates, while cards 1031/1032 varied only the protein and buffer flow rates (see Table 1). A possible explanation for the larger crystals in 1033/1034 could be that the very slow flow rate of the carrier fluid in 1033/1034 created non-uniform, large plug volumes leading to more protein available in each plug for crystal growth. 

As seen in Figure 5, some lysozyme crystals grew wide along the channel width direction, which could cause crystal deformations. Since this experiment focused on crystal growth and not diffraction quality, the effect of this on data quality is unknown. In μg PCG’s Shuttle and Mir days (late 1980’s to early 2000’s), it was common to grow very large crystals, but currently, due to the availability of highly collimated and powerful synchrotron beam lines, it is now not uncommon to diffract useful data from much smaller crystals (25-10 μm) [39-42]. With the Plug Maker™ method, depending on the crystal morphology, it is possible to grow crystals of at least ~150 μm wide (channel width is 200 μm) before deformations could occur, yielding crystals that are certainly large enough to obtain high quality diffraction data. Secondly, crystal size can easily be controlled by altering the ratio of protein/buffer/precipitate through changes in the machine’s flow rate parameters. 

#### Thermolysin

**Figure 6:**
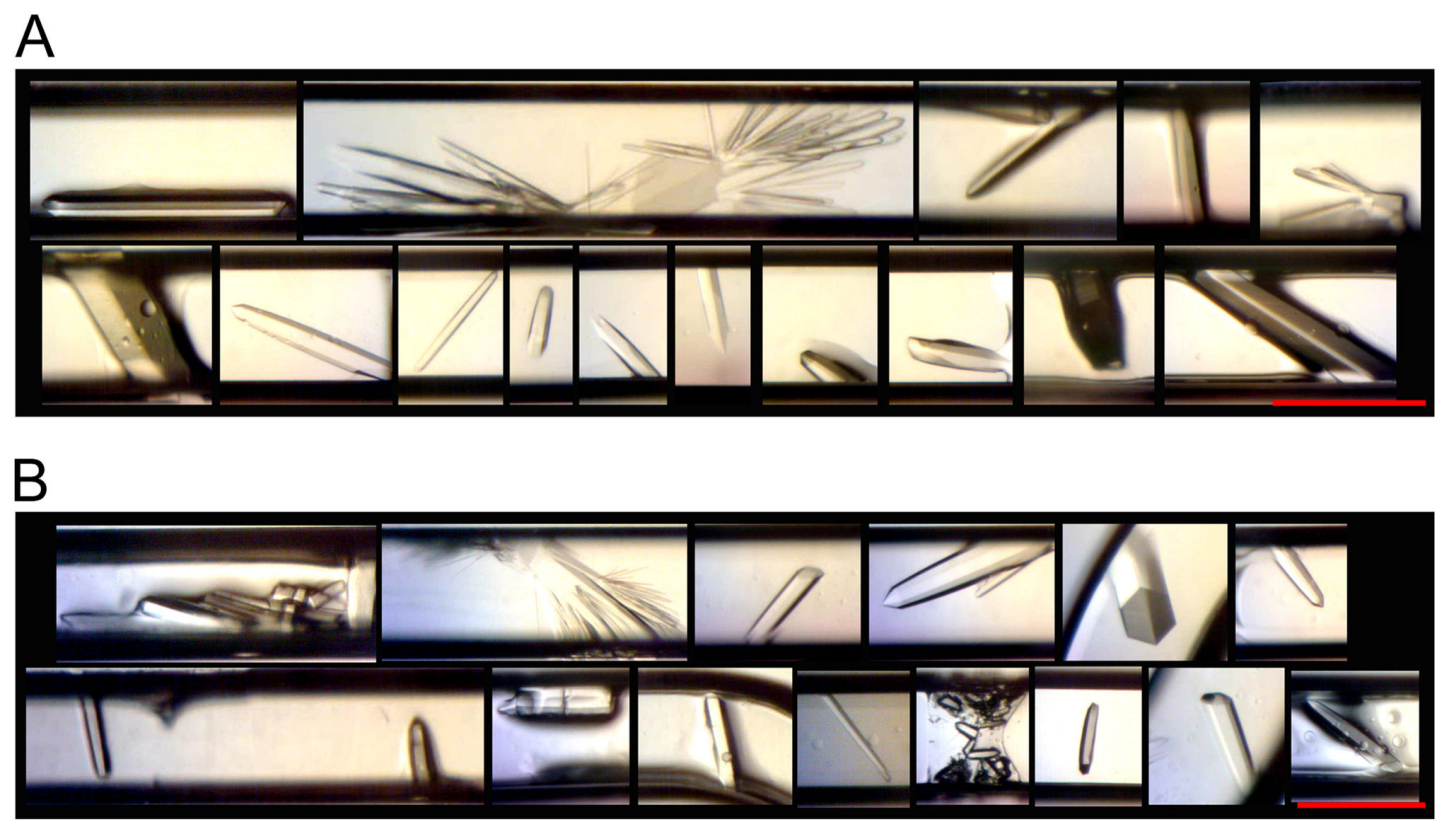
Examples of Thermolysin Crystals. (A) Returned from microgravity and (B) ground control crystals. Red bar indicates 200 μm.

All thermolysin cards had hexagonal rod shaped crystals present (Figure 6), with microgravity cards 1007 and 1013 displaying about 20 single crystals each, 1011 had 14 crystals and 1009 had six. The number of crystals found in the respective ground controls was less, with 1014 (ground control for 1013) having the most at 12 crystals. The largest crystals were seen in microgravity cards 1011, 1012, 1013, and 1014 at ~200+ μm. All drops were clear for both microgravity and ground control cards. 

The parameters for 1013/1014 that created the most crystals in both flight and ground controls (22 and 12 crystals, respectively) had the flow rates of all four channels fixed, creating one long plug in both cards. It is interesting to note that these parameters were also used on lysozyme, but with dissimilar results, underscoring the importance of a large variety of sample conditions for optimal PCG results. 

A search of the literature shows that thermolysin μg PCG was performed twice before on unmanned 8 and 14 day flights [43,44]. Both missions yielded crystals that were almost 10X larger than those grown in their 1g controls, but diffraction for the microgravity samples were not as good as the 1g crystals. Our microgravity and ground controls crystals were of smaller size (due to less volume of protein) and were grown for a longer amount of time, with a different technique, and another precipitant than previously. Because of these differences, it will be interesting on future flights to determine if our crystals will have the same diffraction quality as these unmanned missions.

#### Xylanase

**Figure 7:**
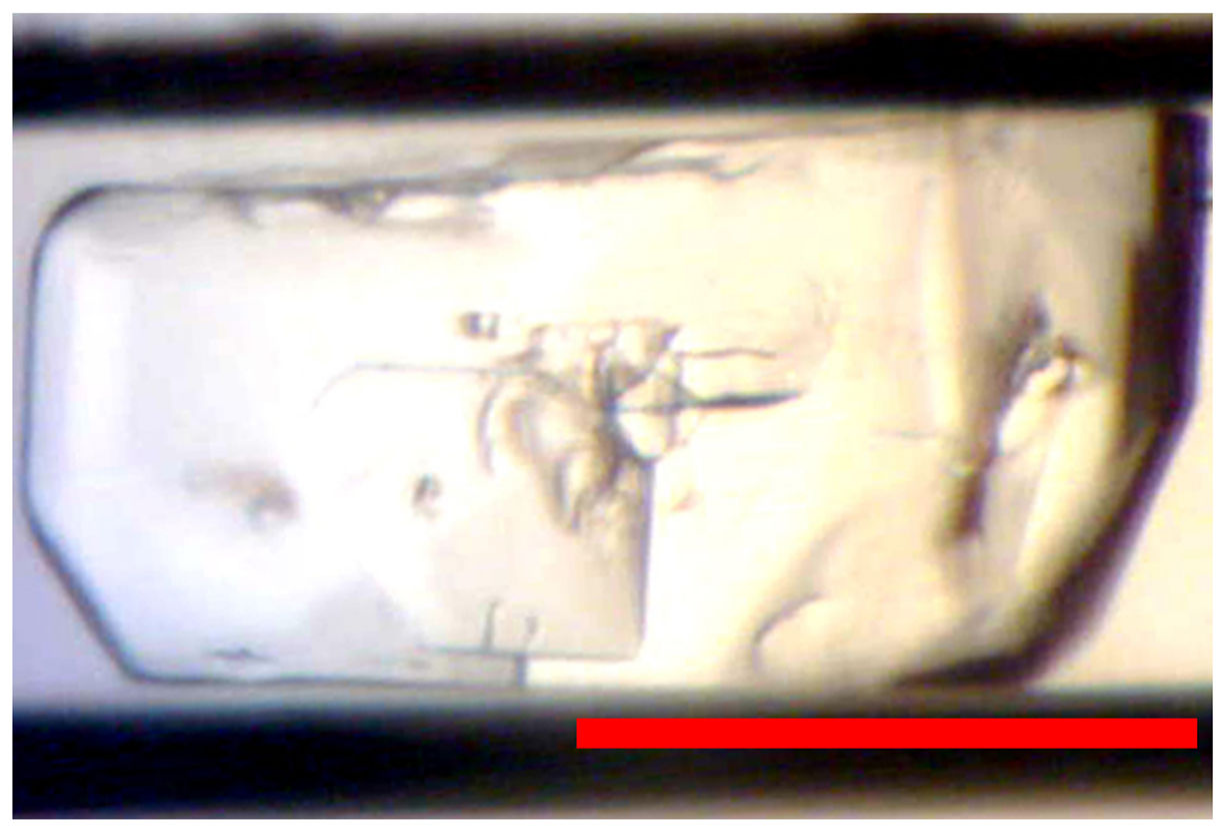
Xylanase Crystal. This was the only xylanase crystal found in the returned microgravity card. Red bar indicates 200 μm.

Only one large irregularly shaped xylanase crystal was found in microgravity card 993 (Figure 7). No crystals were seen in any ground control cards. The crystal is ~250 μm x ~190 μm and appears to be twinned, growing from a common nucleus with one side growing around the other. All other drops in the microgravity and 1g PCG cards were clear, with some phase separation and no visible precipitate.

A report of xylanase crystals grown in microgravity appears at least once, but it is unclear what the morphology of those crystals were [45]. Our results are possibly because of under optimized conditions or intolerance to freezing. 

## Summary and Conclusions

The goal of this experiment was to test the ability of growing protein crystals in microgravity using a COTS high throughput method. Overall, our results were very promising with 16 of 25 microgravity containing crystals, compared to 12 of 25 of the ground controls. Due to the very favorable results for the apo-hPPAR-γ LBD we will continue to test this protein as well as other nuclear receptors using the PlugMaker™/CrystalCard™ µg PCG method to determine if we can improve our existing 3D models. 

Due to unclear on-orbit microscope pictures, we are only absolutely certain that one card contained protein crystals grown in microgravity. Yet, because the small possibility of crystallization occurring during the many temperature variations that occurred during their transportation from the ISS to the lab, we are confident our goal has been achieved. As for the samples that did not grow crystals, this could be due to several variables, such as optimizing the protein starting concentration, exploring the time required for μg crystal growth using this method or providing stable temperature control. 

Without diffraction information, we are not absolutely certain that the crystals observed is this study are indeed from protein, but could be from reagents found in the buffer and/or precipitant. That being said, with our experience in growing apo-PPAR-γ LBD [46,47] and lysozyme crystals, we are certain that the morphology of the crystals found in this experiment is the same as those we have obtained diffraction data from in the past. The morphology of thermolysin is also the same as others have observed from this well-known standard. The odd morphology of xylanase is the only real uncertainty, as it does not match any previous crystal structure we have seen.

It is possible that buoyancy driven convection may already be reduced in 1g microfluidic devices [8,48,49]. The dimensionless Grashof number is a ratio between buoyant and viscous forces in a fluid, providing an approximate scale of buoyancy driven convection:

$${\text{G}}_{\text{r}}{\text{=L}}^{\text{3}}\beta_{1}\Delta \text{c}gv^{\text{-}2}$$

where L is characteristic container length (cm), Δc is the concentration difference (mg/cm^3^), β_1_ the solutal expansion coefficient (cm^3^/mg), *g* is the acceleration due to gravity (cm/s^2^) and ν is the kinematic viscosity (cm^2^/s). A small value for G_r_ predicts a decreased influence of convection on a system and it can be seen from the equation that there are three possible ways to do this: increase viscosity (ν), decrease the acceleration due to gravity (*g*) or, as in microfluidic devices, decrease sample volume (L). While methods capitalizing on varying the equation’s coefficients have shown promise [8,50,51], calculation of the Grashof number depends on initial system values and may not always correctly estimate what is occurring during the dynamic and complex process of protein crystal growth. After creating a computational simulation using the Grashof equation demonstrating that smaller drops may lead to reduced convection, Carter et al. had equivocal results when comparing the resolution and mosaicity of 1g crystals grown in small (nL) and large (μL) volume drops. Another group growing triose phosphate isomerase crystals using agarose to increase viscosity failed to grow better quality 1g crystals than those grown in an identical system in microgravity [53]. Most important to this experiment and for us a marker of success since this protein is not a standard and has never been grown in microgravity before, was that there were 17 μg apo-PPAR-γ LBD crystals compared to 4 in the 1g cards. While this experiment needs repeating and quality of diffraction determined, having more crystals than were grown on the ground demonstrates a possible advantage to using this system in microgravity. Future research is needed in determining if microfluidic devices can consistently mimic the benefits of μg PCG or even possibly enhance it.

Overall, by combining Protein BioSolutions’ Plug Maker™, CrystalCards™ and NanoRacks’ NanoLab, this method creates the ability to use a small quantity of protein to evaluate hundreds of microgravity crystal growth conditions. Additional advantages to this approach include: 1. the sample is enclosed within the CrystalCards’™ channels, alleviating two previous microgravity PCG difficulties of fluid containment and the required layers of biohazard safety restriction. 2. Retrieval of crystals is easily performed by removing the card seal, or if the investigator desires, X-ray diffraction data can be collected while the crystal stays in the card.

In light of these encouraging results, with future modifications such as active temperature control and automated documentation systems, we believe this method will provide new opportunities for researchers to use microgravity protein crystal growth as a tool for creating improved or novel models.
